# Using maternal sepsis patient journeys to map and prioritise barriers to quality maternal healthcare in Malawi: a multidisciplinary stakeholder consultation workshop

**DOI:** 10.1080/16549716.2025.2451467

**Published:** 2025-02-03

**Authors:** Yamikani Chimwaza, Dalisto Ndaferankhande, Leonard Mndala, Chifundo Ndamala, Emily Lifa, Mercy Machilika, Esther Mwagomba, Bernard Dossie, Meliya Kwelepeta, Bertha Maseko, David Lissauer, Maria Lisa Odland

**Affiliations:** a Maternal and Fetal Health Group, Malawi Liverpool Wellcome Clinical Research Programme, Blantyre, Malawi; b Department of Women’s and Children’s Health, Institute of Life Course and Medical Sciences, University of Liverpool, Liverpool, UK

**Keywords:** Patient journey, delays, barriers, prioritisation, maternal sepsis, pregnant and postpartum women, quality of care, maternal healthcare, Malawi

## Abstract

**Background:**

Malawi has made progress in improving access to maternity care services, shifting the focus to quality of care as an essential determinant of maternal health outcomes. However, no effective mechanisms exist to use patients’ experiences of care at health facilities to inform and improve the quality of maternal healthcare.

**Objective:**

To use maternal sepsis patient journeys in a workshop with maternal health stakeholders to identify and prioritise barriers in care and recommend interventions to improve maternal healthcare quality in Malawi.

**Methods:**

In February 2024, in Blantyre, Malawi, using a modified nominal group technique, 28 stakeholders reviewed the patient journeys of three women hospitalised at Queen Elizabeth Central Hospital, who had sepsis after childbirth. Patient journeys narrate events experienced within a healthcare system in the patient’s words. In a multiframework approach (Four Delays, Respectful Maternity Care, and WHO Quality of Care), stakeholders identified and prioritised barriers to care and recommended interventions to improve the quality of maternal healthcare. Content analysis of the workshop data linked barriers with stakeholders’ suggested interventions.

**Results:**

Nineteen barriers identified included various delays in receiving care, mistreatment by healthcare providers, and suboptimal quality of care. Stakeholders found patient journeys valuable and insightful for identifying gaps in the quality of care and promoting sepsis awareness among healthcare workers and the public.

**Conclusions:**

Patient journeys are a novel tool for capturing the experience of care in Malawi. They have the potential to guide strategic improvements in maternal healthcare quality and ultimately reduce maternal morbidity and mortality.

## Paper Context

- **Main findings:** In this workshop, we observed the benefit of incorporating patient journeys into maternal healthcare prioritisation exercises. This approach offers valuable insights into patients’ experiences, providing honest perspectives on the entire care journey. It could complement standard hospital audit data, enhancing the impact of quality improvement initiatives.
- **Added knowledge:** Serious delays in receiving appropriate care, mistreatment from healthcare providers, and suboptimal quality care are the experiences of maternal sepsis patients in Malawi.
- **Global health impact for policy and action:** Prioritisation exercises, informed by the patient journey, could guide strategically targeted interventions and a multifaceted approach to improve the quality of maternal healthcare in Malawi and globally.

## Background

Infection, especially sepsis, is a significant cause of maternal, neonatal and child morbidity and mortality [1]. Pregnant and recently pregnant women quickly develop infections and are particularly prone to rapid progression to sepsis [2]. Maternal sepsis is a life-threatening condition defined as organ dysfunction resulting from infection during pregnancy, childbirth, postabortion, or the postpartum period [3]. Globally, maternal sepsis is the third most common cause of maternal death after postpartum haemorrhage and preeclampsia [4,5]. Maternal infections are the number one cause of maternal mortality in Malawi, contributing to 24.8% of all maternal deaths [6].

Malawi has made steady progress in improving access to maternity care services, with high rates of women accessing antenatal care (97%) and over 96% of the women having facility-based births attended by a skilled healthcare provider [7]. The increase in access to care now shifts the focus to the quality of maternity care, which becomes a more important determinant of maternal health outcomes in this setting [8]. Malawi has been highly committed to improving the quality of maternal, newborn and child services across the health system. However, the health system is complex, and care is often suboptimal, fragmented, and of poor quality [9–11]. This is particularly true for maternal care, especially when managing time-sensitive, life-threatening complications such as maternal sepsis. High-quality care is meant to be safe, effective, timely, efficient, equitable, and people-centred [9]. When maternity care is high-quality, more than 147,000 maternal deaths can be averted each year globally [9,12,13].

An expanding focus has been placed on the experience of care as an essential indicator of quality [14]. Health facilities in Malawi currently lack effective methods for patients to provide feedback on their care experiences. As a result, healthcare improvement and reform strategies are not benefiting from patient feedback. This could be due to patients not knowing where to share their complaints and feedback or the existing systems being underused and ineffective, resulting in no improvement in healthcare services [15,16]. Strengthening health systems requires a better understanding of care recipients’ experiences and systematically exploring the reasons for suboptimal care with healthcare providers and facility managers in different settings. Identifying barriers to care and developing health system priorities to address these barriers can help to inform quality improvement measures in maternity healthcare. The purpose of this workshop was to engage maternal health stakeholders in the use of maternal sepsis survivors’ perspectives and experiences of care to map and prioritise barriers in maternal healthcare and recommend corrective and preventive interventions for improving maternity care quality in Malawi.

## Methods

### Study design

This participatory study utilised a modified Nominal Group Technique (NGT) [17–19] for a structured approach to group breakout sessions during a workshop. In these sessions, stakeholders were asked to identify delays and prioritise barriers to maternal healthcare based on the narratives of patients who experienced maternal sepsis. We modified the NGT approach by including additional steps for brainstorming corrective and preventive solutions or interventions with full participation from all stakeholders. We used the NGT as a well-validated, practical approach to encourage the full involvement of stakeholders, consider all views equally, and build a consensus on prioritising barriers to quality maternal healthcare.

### Setting

The workshop occurred in February 2024 in Blantyre, Malawi’s commercial capital. The venue was conveniently located in the central business district, making it easy for all stakeholders to access.

### Participants

Invited stakeholders represented those with direct contact with maternal patients as healthcare providers from all levels of service delivery: the primary care level (nurse midwives and clinical officers from health centres), community healthcare level (safe motherhood coordinators), tertiary care (nurse midwives, clinicians, nurses in charge of maternity wards and operating theatre, and private sector (Obstetrician Gynaecologist specialists). We also invited representatives from the district health management team and hospital management for their input on maternity care’s structural, policy-related and managerial aspects in the health system. We involved representatives from the Malawi Liverpool Wellcome Programme Patient and Public Involvement (PPI) [20] group and the Queen Elizabeth Central Hospital Ombudsman to provide public and patient perspectives and concerns about the quality of maternity care. All stakeholders were invited by email. Stakeholders were purposefully selected from the author’s networks to ensure a balanced representation of healthcare provider cadres and geographic areas (within Blantyre city) of practice.

### Data collection

This stakeholder consultation workshop was part of the ‘Understanding and improving the diagnosis of maternal sepsis’ study. This mixed-methods study was conducted from April to May 2023 in Blantyre, Malawi. Using purposive sampling, pregnant, postpartum, and postabortion women over the age of 16 who had sepsis underwent face-to-face interviews after being discharged from the main tertiary and referral health facility in Blantyre, the Queen Elizabeth Central Hospital (QECH). Deidentified interview transcripts were utilised as patient journeys (i.e. the entire sequence of events a patient goes through inside a healthcare system, across providers, from admission to discharge or recovery) [21] for the workshop. In the preliminary phase, the authors purposively selected three patient journeys with varied experiences and causes of maternal sepsis (covering cases of sepsis due to peritonitis, postpartum endometritis, and surgical wound infection). The selected journeys had the most information power, with easy-to-follow storylines and well-elaborated patient-healthcare provider interactions.

### Introduction and explanation (stage one)

The eight-hour multistakeholder workshop began with a plenary session, where the facilitator (YC, a qualified researcher) provided stakeholders with an overview of the epidemiology of maternal sepsis and the general findings of the qualitative interviews. This was followed by a brief introduction to the conceptual frameworks (Four Delays, Respectful Maternity Care, and WHO Quality of Care) for the idea generation component of the workshop.

A multiframework approach was necessary to identify delays and barriers to quality care for mothers with sepsis. First, we modified the Three Delays Model [22] to examine the complete course of maternal sepsis by adding a fourth delay focused on the post-sepsis period. Post-sepsis syndrome refers to persistent physical, medical, cognitive, and psychosocial problems following sepsis. The recovery process from sepsis can take months or even years, with long-term consequences [23]. There are potential risks of delays in detecting and addressing post-sepsis sequelae that women may suffer once they are discharged from the hospital. Therefore, the Four Delays (4D) framework examined delays in (1) seeking, (2) reaching, (3) receiving, and (4) managing post-sepsis complications with appropriate and quality health care. Respectful and inclusive care is crucial to quality maternity care [24]. The domains of the Respectful Maternity Care [25] framework can be used to assess each patient’s journey to establish whether a health facility and its health providers meet women’s expectations of respectful and inclusive care [26]. The RMC domains are (1) no harm or mistreatment; (2) information, choice, and preference; (3) privacy and confidentiality; (4) dignity and respect; (5) equality and equity, no discrimination; (6) high-quality care; (7) liberty and autonomy, no arbitrary detention; (8) child with parents or guardians; (9) child identity and nationality from birth; and 10) adequate nutrition, clean water (14). Last, the third framework used was the domains of the WHO Quality of Care [24], namely, (1) evidence-based practices for routine care and management of complications, (2) actionable information systems, (3) functional referral systems, (4) effective communication, (5) respect and preservation of dignity, (6) emotional support, (7) competent, motivated healthcare providers, and (8) essential physical resources available [14,15]. The WHO QoC framework is well suited for evaluating each patient journey by assessing the overarching capacity of the health system (following a structure-process-outcome model) while focusing on the dimensions related to the provision and experience of care [24].

### Patient journey review (stage two)

Stakeholders were then randomly allocated into four groups (6–8 stakeholders per group) and were provided with flipcharts, writing materials, sticky notes and group sitting arrangements. A volunteer in each group read aloud the patient’s journey. Everyone in the group was requested to individually list any delays or apparent barriers from any constructs of the 4D, RMC, and WHO QoC frameworks that were identifiable in the patient’s journey. Everyone wrote down the ideas on sticky notes. Hand-out copies of all three frameworks were provided to each group for use during this stage, which took approximately 45 minutes.

### Round-Robin recording ideas (stage three)

A representative in each group led the stakeholders in sharing and posting their ideas on a flipchart until all the ideas were exhausted. When ideas or concepts were similar, stakeholders discussed and agreed on the wording. Any new ideas that arose with the deliberations were added to the flipchart. This stage lasted approximately 20–30 minutes.

### Group discussion (stage four)

Through in-depth group discussions, stakeholders were guided to convert each delay into a barrier based on agreed-upon root causes or contributing factors. Delays were then renamed to the identified barrier. Each stakeholder within the group had an opportunity to contribute, suggest new ideas for discussion, and combine items into categories. This stage took approximately 45 minutes.

### Ranking and prioritisation (stage five)

Each group member ranked and rated the barriers from the compiled list. The group then collectively used the ranking (most to least significant) and rating systems (priority number one to however many were identified) to reorganise the sticky notes of barriers on the flipchart into their agreed-upon order of priority. This stage took approximately 15 minutes.

### Touchpoints, corrective and prevention interventions (stages six, seven and eight)

Stakeholders were asked to identify where the intervention was required in the patient’s journey (a touchpoint). A touchpoint in the patient’s journey is any point at which a patient interacts with health services and presents an opportunity to improve or compromise the patient’s experience [27]. Examples of touchpoints could be at the admission desk in the labour ward, during preoperative assessments, in the immediate postpartum period, and so on. For each identified barrier, corrective intervention (intervention(s) that might have improved the severe maternal outcome at the touchpoint) and preventive intervention (intervention(s) that could prevent future occurrences of the same severe maternal outcome) were proposed by the stakeholders. This stage took approximately 60 minutes.

### Final consensus

**Figure 1.**
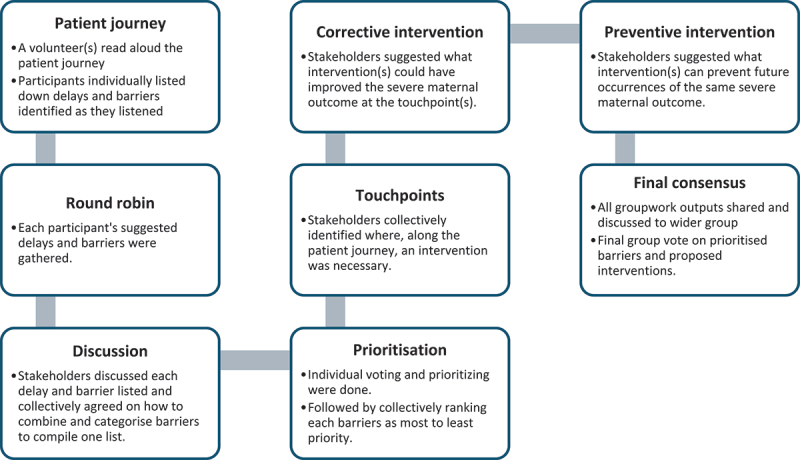
Flow diagram of the modified nominal group technique for identifying delays, prioritising barriers, and proposing interventions for maternal sepsis.

Once the breakout sessions were completed, the wider group was reconvened for a presentation of group work. One member from each group read out the barriers and how they were prioritised and justified the proposed corrective and preventive interventions. The facilitator wrote each group’s results on a projected screen for all participants. After a lengthy discussion and a final group vote, the top four barriers and proposed interventions to improve care for maternal sepsis in Malawi were finalised. A facilitator assistant took minutes to capture key discussion points during this stage. Figure 1 shows a flow diagram of the modified nominal group technique for identifying delays, prioritising barriers, and proposing interventions for maternal sepsis.

## Data management and analysis

After the workshop, we collected all written group work outputs, such as flipcharts and sticky notes, and stored them at Malawi Liverpool Wellcome Trust. Through content analysis, we analysed the data from the flipcharts, sticky notes, and facilitator assistants’ minutes to identify the intentions, focus, and trends in categorising delays and prioritising barriers from the stakeholders’ group work and discussions. We reduced the data (presence of delays, barriers identified, and interventions suggested) to examine the occurrence of explicit terms and concepts. We used the stakeholder’s collective list of delays and barriers as predefined codes or concepts to categorise and organise the data. Barriers were prioritised and coded based on the frequency with which a concept appeared in the data. Barriers were then aligned to proposed corrective and preventive interventions mapped by stakeholders for each patient journey. The data were interpreted, and conclusions and generalisations were drawn from explanations, reasoning, and conversations captured during the broader group discussion. Since we did not have informed consent from stakeholders to record direct quotes, we made inferences from the discussions.

## Ethical considerations

This stakeholder consultation workshop was part of a study protocol called ‘Understanding and Improving the Diagnosis of Maternal Sepsis,’ which was approved by the Kamuzu University of Health Sciences Research Ethics Committee (P.10/22/3793) and the University of Liverpool Central University Research Ethics Committee (UoL001719). The stakeholders gave verbal consent to publish the workshop material.

## Results

Twenty-eight stakeholders participated in the workshop in Blantyre. Table 1 shows the breakdown of the stakeholders.

**Table 1.** Summary of participants for the stakeholder consultation workshop in Blantyre.

Cadre	n	Facility/Organisation
Nurse/midwife	8	Central Hospital
Clinician	2	Central Hospital, Health Centre
In-charge of Ward/Health centre	5	Central Hospital, Health Centres
Safe Motherhood Coordinator	5	Urban and rural health facilities
Health Management Team member	1	District Health Office
Hospital administrator/Director	1	Central Hospital
Ombudsman	1	Central Hospital
Patient and Public involvement representative	2	Local community
Maternal health researcher	3	Research Institution
Total	28	

**Figure 2.**
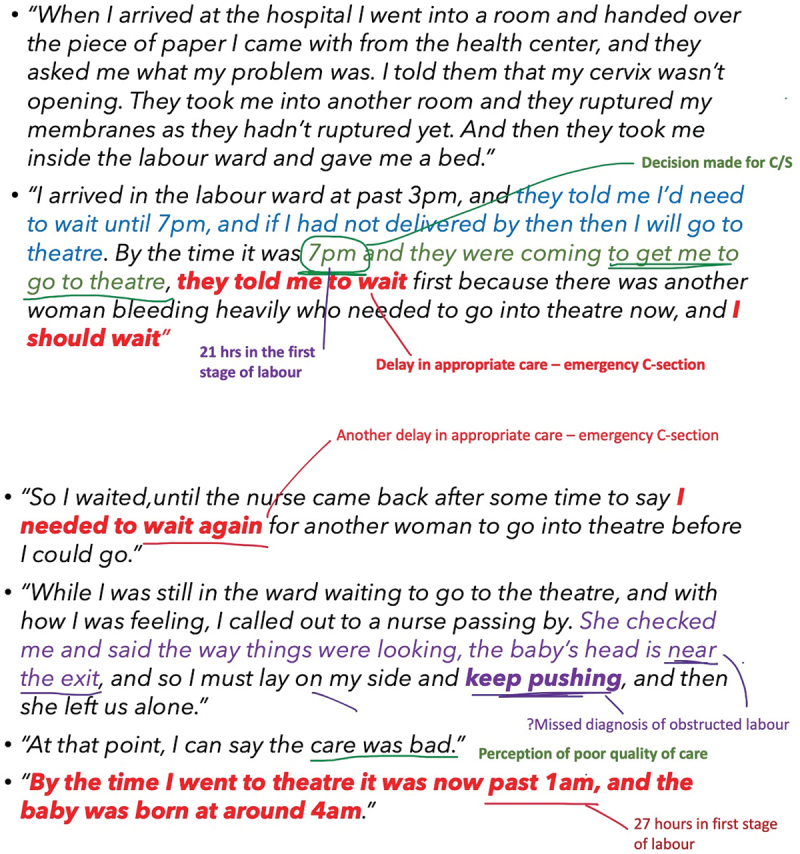
Schematic excerpt from reviewing one patient’s journey and mapping delays and barriers in maternity care.

**Figure 3.**
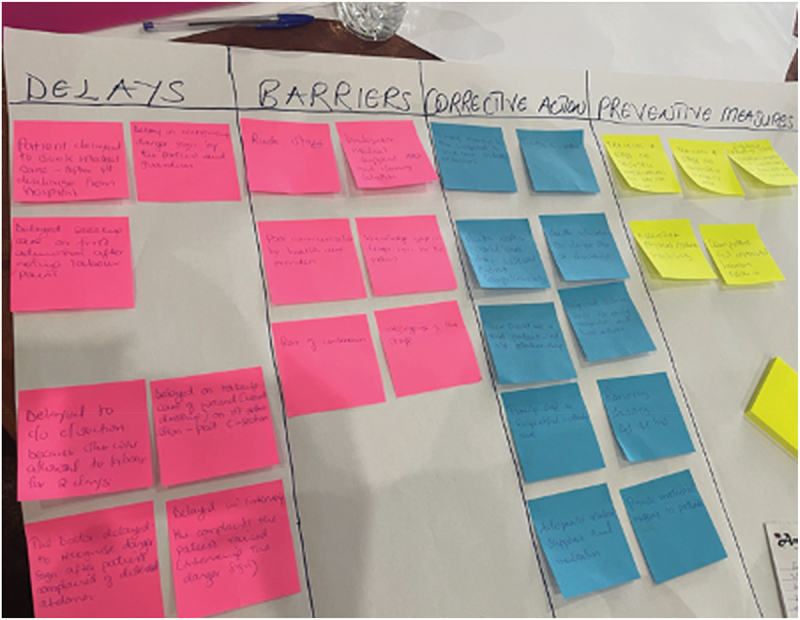
Workshop photo of an example of one of the group’s mapping and prioritisation layouts during the discussion phase.

**Figure 4.**
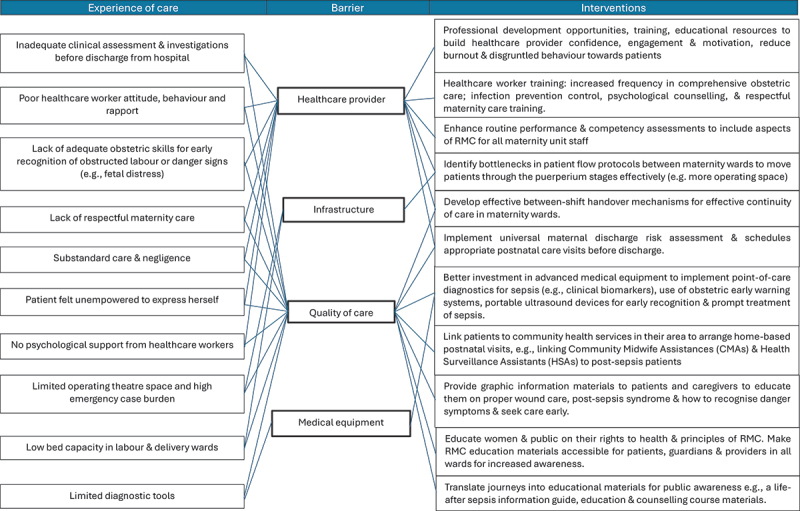
Schematic diagram of maternal sepsis patient experience of care linked to barriers and proposed interventions.

At the workshop’s close, 35 constructs from the 4D, WHO QoC and RMC frameworks were identified from three maternal sepsis journeys. Eight delays from the 4D framework included various types of third delays (delays receiving appropriate care, for example, missed or delayed diagnosis of severe maternal outcome, delayed administration of essential treatment, delayed emergency caesarean section) and one type four delay (delayed post-sepsis management of an obstetric fistula). Two concepts from the WHO QoC framework were identified: ineffective communication and provision of poor care (i.e. lack of psychological counselling to a patient needing professional support). Four components of RMC were identified, namely, lack of autonomy, undignified care, verbal abuse and disrespect. Overall, stakeholders combined and categorised 19 barriers: six barriers related to healthcare providers, three barriers related to infrastructure, one barrier related to medical equipment, and six barriers related to quality of care. The stakeholders identified touchpoints along each patient’s journey where four corrective interventions were necessary, and 19 preventive interventions were proposed. Figure 2 shows how stakeholders comprehensively reviewed patient journeys to identify delays and barriers. Figure 3 shows a workshop photo of the mapping and prioritising exercise, and Figure 4 shows a schematic diagram of experiences of care that were linked to barriers and interventions.

### Priority setting

The stakeholders selected ‘healthcare provider’ barriers as the highest priority. Stakeholders pointed out that delays in referrals from the health centre to the tertiary hospital, delayed emergency caesarean sections (CS) following signs of fetal distress, and missed diagnoses of complications were all healthcare provider-related barriers. They noted that these delays stemmed from healthcare workers either lacking or failing to apply the emergency obstetric skills necessary for identifying obstructed labour, which requires urgent life-saving interventions for both the mother and the fetus. Furthermore, they highlighted the case of a woman referred to QECH for fetal distress, who reported being left waiting for several hours before her caesarean delivery. Lastly, the stakeholders remarked that inadequate clinical assessments and investigations before discharge might have led to one woman returning to QECH just three days post-delivery with a severe surgical wound infection, which could have been identified with a thorough discharge evaluation, especially since this woman expressed feeling unwell and exhibited early signs of infection before she was sent home.

The second priority was ‘infrastructure’ barriers related to limited operating theatre space and insufficient bed capacity to manage the high burden of obstetric emergencies. The stakeholders pointed out numerous touchpoints along the patient journeys where a surgical intervention could have averted the severe maternal outcomes experienced. They noted in one patient’s experience that women had to wait for an emergency CS if another woman was considered more urgent. More operating theatre space would have averted the delay. Some nurses shared examples of the realities where low bed capacity in labour and delivery wards has caused a backlog of patients all needing immediate care. This leads to congestion in the admissions area, where coordinated clerking and assessments cannot be done adequately. During the group discussion, the district and hospital management stakeholders emphasised that the lack of a district hospital in Blantyre is a critical barrier to improving the management of obstetric emergencies and mitigating barriers to comprehensive maternity care.

The third priority was the ‘quality of service delivery’ barrier. During the workshop, stakeholders deeply empathised with the women’s experiences. Some interactions between healthcare providers and patients highlighted poor attitudes and behaviours from healthcare providers. Stakeholders noted that the care these women received lacked the principles of respectful maternity care. This was clear in the patient experiences, where women reported delays in receiving antibiotic treatment due to a provider’s oversight, instances of nurses being rude to patients in pain, disrespectful and undignified treatment towards women, and ineffective communication. The women felt unable to voice their concerns, and healthcare providers did not offer any psychological support. Overall, stakeholders raised concerns about the mistreatment and the suboptimal quality of care. Many nurse-midwives and clinicians pointed out many apparent deviations from standard care practices. They regarded the provision of care as substandard, with instances of negligence noted in these three patient journeys.

The fourth priority was ‘medical equipment’ barriers. The stakeholders identified this barrier in the patient journey, noting that a quicker diagnostic tool for severe intra-abdominal infection could have helped detect the onset of sepsis in one woman. She had to wait several days for an abdominal x-ray. At the same time, stakeholders remarked that point-of-care tests or early warning systems could have identified her condition sooner, leading to earlier intervention before she developed peritonitis, which resulted in her admission to the intensive care unit for several days.

After prioritising barriers, the stakeholders identified touchpoints during patient journeys where corrective and preventive interventions were necessary. For the three patient journeys, the stakeholders identified that corrective interventions could have been as follows: (1) Earlier referrals from the health centre to the tertiary hospital were required for patients with poor progress in labour. (2) Identifying alternative operating spaces and mobilising additional staff are warranted to manage the high burden of obstetric emergencies. (3) Advanced diagnostic blood tests, point-of-care ultrasound, or exploratory laparotomy could be used to detect postnatal infections sooner. 4) Enhanced intrapartum maternal and foetal monitoring during waiting times in labour ward admission would have detected fetal distress much sooner.

To prevent future occurrences of the same sepsis-related maternal outcomes that these survivors experienced, stakeholders proposed a range of preventive interventions for healthcare providers, infrastructure, medical equipment, quality of service delivery, and the community. Figure 4 displays a schematic diagram linking the experiences of care to barriers and proposed interventions as agreed by stakeholders. Table 2 presents excerpts of patient quotes that stakeholders connected to each delay, barrier, priority ranking, and touchpoint in the journey.

**Table 2.** Excerpts from three patient journeys with delays, barriers, priority ranking, and touchpoints identified by stakeholders.

Delays/RMC/QoC domains	Quotes	Barriers	Priority ranking	Touchpoint
Delay 3: Delayed referral from health centre	‘When I arrived at Zingwangwa health centre they assessed me and said my cervix was 4 cm. Then when they checked me at 1am I was still 4 cm. And then at 11am when I was checked I was still 4 cm. Then at 2pm the nurse told me my cervix is still not opening and that I needed to go to Queens for proper management.’	**Healthcare provider**: Lack of obstetric skills and equipment for early recognition of obstructed labour	1	Labour ward at health centre
Delay 3: Delayed emergency c-section	‘I arrived in the labour ward at past 3pm, and they told me I’d need to wait until 7pm, and if I had not delivered by then then I will go to theatre. By the time it was 7pm and they were coming to get me to go to theatre, they told me to wait first because there was another woman bleeding heavily who needed to go into theatre now, and I should wait. So, I waited, until the nurse came back after some time to say I needed to wait again for another woman to go into theatre before I could go. By the time I went to theatre it was now past 1am, and the baby was born at around 4am.’	**Infrastructure**: Limited operating theatre space, and high patient burden (too many emergency cases)	2	Labour ward at tertiary hospital
Delay 3: Delayed diagnosis of intra-abdominal infection	‘It started after her caesarean section, when they removed her catheter, we’d find that her urine was just leaking out. And then when they put the catheter back is when she’d start complaining of pain in the abdomen. It was as if there was a mass. It was difficult for the nurses to understand and believe us, but I was certain that something was going on. But I didn’t want to let go of this complaint. It got very bad, to the point that when she took Panadol nothing was changing. No medicine was working. They even started giving her injections for pain, those ones given every 6 hours, but it wouldn’t even last 6 hours for her to be in pain again. Sometimes it would be only 2 hours and then she’d be in pain again. When the x-ray was done, we were told that she had a mass in her abdomen, and she needed to go to the theatre the next day. The next day at 8 am, they were taking her to the theatre; at this point, she was very weak. The nurses even wanted to give her oxygen on the ward but ended up taking her straight to the theatre.’ Guardian	**Medical equipment**: Limited diagnostic tools	4	Postnatal ward at tertiary hospital
Delay 4: Delayed repair of obstetric fistula	‘What has changed is [breaks down crying], during delivery they cut my bladder, but they sowed it back together. So, when I’m having sex with my husband [pauses crying and catching her breath] … during sex when you are supposed to have vaginal discharge coming out, urine starts coming out. And this still happens till now.’	**Quality of service delivery**: No access to vesicovaginal repair service	3	At patient’s home and community
Delay 3: Delayed decision to perform c-section	‘Yes, I had to wait, because inside labour ward there were other patients who had just delivered, and we had to wait until they have been removed and taken to the recovery room. We also had to wait for those in recovery to rest for about two hours, and then they would go to postnatal ward. I waited and only when patients were moved out is when they gave me a bed. And there they just gave me a bed, they didn’t assess me then, they just told me to wait.’	**Infrastructure**: Low bed capacity in labour and delivery suites. High patient burden	2	Labour ward admission area, at tertiary hospital
Delay 3: Delayed emergency c-section after clinical sign of foetal distress	“They just left me until my water broke. My sister went to call the nurse to come check on me and the baby. When my waters broke, I could see the baby’s poop in the fluid. The nurse told me that the baby had pooped inside my uterus when she checked me out. She also checked my cervix and saw that it had closed again to 3 cm. The nurse told me she had to talk with her co-workers about what to do next and would get back to me with her thoughts. After some time, she came back to tell me I had to have surgery. My water broke early in the morning, around 1 am. From then on until 5 p.m., when I went to the theatre, I waited. It feels like I waited in the labour ward for 12 hours. Because I was told in the morning that I was fourth in line to go to the theatre. But I was stuck in a waiting mode. They would come to check my cervix and blood pressure and then leave. After that, someone else would do the same thing and leave. They kept telling me I was number 4 and that I could go in whenever it was free.	**Infrastructure**: Limited operating theatre space. **Healthcare provider**: Insufficient care after clinical sign of foetal distress	2	Insufficient care after clinical sign of foetal distress
Delay 3: Delayed administration of antibiotic treatment	‘Some nurses, especially those who gave medicine in the morning, would bring the medicine that needed to be injected and leave it there, saying they’d be back to do it. When they came to leave the medicine at 5 a.m., the person who was supposed to give us the injection would leave for hours and forget about us. When someone else comes along, we’d ask why they left this injection and if they weren’t going to give us an injection. And only then would they give us the shot. This would happen most of the time at night. It took place several times.’	**Quality of service delivery**: Substandard care and negligence	3	Gynaecology ward at tertiary hospital
Delay 3: Missed diagnosis of a complication	On day 3 post-delivery before I was discharged from the hospital, the doctor was reviewing me they noted that my abdomen was distended and hard. I even asked them why my stomach was so big and hard like that. Even the doctor’s facial expressions that showed they, too, were puzzled and wondering, and the Dr said, ‘Maybe it’s because your baby was big, so your uterus was also very enlarged; it just means it will take some time for your uterus to get small again.’ This was my first pregnancy and first surgery, and I didn’t know what to expect or anticipate. I really felt like my health was somehow compromised after the c-section. I believe I was healthy and fit throughout my pregnancy. I thought maybe that was what happen happens to people after a c-section. On January 1, 2023, my mom came to check on me. She used a cotton ball to clean my incision, but the whole ball got wet with pus coming from the incision. My thoughts at the time were, maybe there was a mistake that happened at the hospital. I told myself something was wrong, but I’ll be fine. Even though they never said it, the people at the hospital knew something was wrong when they discharged me. I believe the problem began at the hospital, while I was there, and specifically during the c-section. My mom called my sister right away to come over. When my sister arrived, she said the whole bedroom smelled awful and showed me a bunch of big black flies in the room and around me. My sister took me back to QECH. When I arrived, the nurse saw the state I was in and quickly took me to her office. I didn’t need to stand in line. The nurse opened the incision and began squeezing and clearing the pus while another nurse quickly called for a doctor to come help me. I don’t remember what the diagnosis was, but when the doctor saw me, she said the wound was still fully intact, and the infection wasn’t very deep.”	**Healthcare provider**: Inadequate clinical assessment and investigations before discharging patient.	1	Gynaecology ward at tertiary hospital
RMC: Verbal abuse RMC: Undignified care	“There were some nurses I got along with well who were understanding and helpful when I was in pain or had issues. Others were rude, just rude. There was one day when I wasn’t feeling well, so I asked for some medication. After more than an hour a different nurse approached me to say ‘In the hospital we don’t beg for medicine, it’s like you are undermining the professionals who are taking care of you,’ when I asked if I was wrong to say something or ask for help I was told ‘you see, that’s exactly why you won’t be helped because it’s like you are argumentative and needy.’	**Quality of service delivery**: Poor healthcare worker attitude and behaviour. Lack of respect and preservation of patient’s dignity	3	Gynaecology ward at tertiary hospital
RMC: Undignified care RMC: Disrespect	“The dressings they put on me got soiled, causing me pain. When I went to tell the nurse that the dressings were wet, they responded, ‘Oh no, you’re smelling, go and bath. When, in fact, the problem was the pus from the wound and not because I hadn’t bathed. So, no one will receive that kind of talk well. Because I knew the problem came from the wound and the soiled dressings. Communication. You nurses should speak to us better. We know you also have your own problems.’	**Quality of service delivery**: Lack of respectful maternity care	3	Gynaecology ward at tertiary hospital
QoC – Ineffective communication RMC: Lack of autonomy	When I look back, I could sense there was something wrong in my body, something was not right. I realised that maybe I made a mistake because while I was in hospital, I didn’t say anything, all the symptoms I was feeling at the time; maybe I would’ve been helped before things got as bad as they did.”	**Quality of service deliver**y: Poor rapport between patient and healthcare worker. Patient felt unempowered to express herself.	3	Gynaecology ward at tertiary hospital
QoC – Provision of care: Lack of professional psychological support for patient during hospitalisation	She was transferred to the surgical ward from the labour ward high-dependency unit. In the surgical ward there were many challenges, she wasn’t walking, and she had many concerns and many worries. I had to keep encouraging and giving her all the support.” Guardian	**Quality of service delivery**: No psychological support from hospital staff	3	Surgical ward at tertiary hospital

## Discussion

At a workshop, sepsis patient journeys were used for maternal health stakeholders to comprehensively identify barriers to providing timely, respectful, and quality maternal healthcare in Malawi. After discussion and consensus, the stakeholders described the patient’s experiences as journeys marred by significant delays in receiving appropriate care at the facility level, compounded by mistreatment from healthcare providers while receiving suboptimal quality care.

### Healthcare provider factors

Healthcare providers in Malawi have repeatedly highlighted various health system failures in matching supply and demand that have clear negative implications for health workers and those directly impacting the care of women [28]. Staff shortages, inadequate skill mixes to address obstetric complications, and exhaustion due to excessive workloads are some of the challenges health workers have reported experiencing in maternity wards [28,29]. Healthcare providers have demanded more supervision and performance feedback to stay motivated, but their needs still need to be met [30,31]. Additionally, when healthcare providers acknowledge their work and have access to more training opportunities, they are more satisfied and motivated [28,32]. Workshop stakeholders suggested regular refresher training and skills workshops on comprehensive obstetric care training to keep rotating staff updated on required skills in maternity wards. Stakeholders also mentioned that healthcare providers need more professional development opportunities and educational resources to help them feel more confident, engaged, and motivated, reducing burnout and preventing disgruntled behaviour from being taken out of patients [28].

### Infrastructure factors

From the patient journeys reviewed, stakeholders identified that limited operating theatre space, low bed capacity in labour wards and high patient burden at QECH posed significant barriers to maternal patients receiving appropriate and timely care. At QECH, the main referral hospital in the southern region of Malawi, its maternity wing is overburdened by high volumes of obstetric emergency cases referred daily from over 15 health centres within the district throughout the year. It also serves a large subset of high-risk pregnancies attending outpatient clinics [33]. Stakeholders strongly recommended establishing a district hospital in Blantyre while enabling existing primary- and secondary-level health facilities to manage more operative deliveries, significantly reducing the patient burden at QECH. Infrastructure expansion in LMICs can take many years; instead, repurposing and renovating current infrastructures, such as secondary-level health facilities or available space within a tertiary hospital, could be more immediate options. For instance, adopting a minor operating theatre to suit obstetric requirements using a mobile delivery pack (neonatal incubator, surgical instruments set, spinal anaesthesia medications, uterotonic crash cart, refrigeration for a small number of blood products) could be a customisable solution that health facilities can explore [34]. Better access to emergency obstetric care (including CS) is the ultimate goal of infrastructure expansion. However, it’s essential to ensure the criteria for emergency CS indications are met to prevent the overuse of surgery, which can lead to harm. From 2020 to 2022, there were 89,098 CS deliveries in 33 health facilities across Malawi, resulting in a CS rate of 16.7% among live births, alongside a maternal mortality rate of 3.1 per 1,000 CS. Each CS necessitates careful evaluation since the risk of death associated with CS deliveries was reported to be over five times higher than that for women delivering vaginally (OR 5.60, *p* < 0.001) in Malawi [35].

### Quality of care

As noted in other studies, health providers in low-income and middle-income countries (LMICs) often perform less than half of the recommended evidence-based care actions. For example, only two in five women who deliver in a facility in LMICs are examined within one hour after birth [9]. Approximately one-third of patients experience disrespectful care, short consultations, poor communication, or long wait times [9]. The stakeholders proposed identifying bottlenecks in patient flow protocols between maternity wards to move patients through the puerperium stages (antepartum, intrapartum and postpartum) more effectively. They also recommend establishing effective handover procedures between shifts for better continuity of care in maternity wards. Additionally, they suggested creating universal clinical tools for assessing maternal discharge risk and conducting follow-up checks, enabling earlier recognition of signs and symptoms of post-sepsis syndrome. All stakeholders agreed that there is a need to establish patient feedback and incident reporting mechanisms that feed into the performance and competency assessments for all maternity unit staff, enhancing accountability.

### Medical equipment

The rapidly progressive nature of maternal sepsis makes it challenging to diagnose and treat [36], especially in low-resource settings such as Malawi. In one of the patient journeys, the stakeholders recognised how the absence of more advanced diagnostic tools led to a delayed diagnosis of a severe intraabdominal infection that eventually required repeated surgical interventions and prolonged intensive care unit admission. The poor availability of laboratory facilities and diagnostic equipment is also a barrier to patient assessment and diagnosis, even when providers know the necessary tests. Therefore, the stakeholders strongly recommended increasing investments in advanced medical equipment to implement point-of-care diagnostics and portable ultrasound devices for the early recognition of sepsis in Malawi [37].

### Strengths of this study

By using three theoretical frameworks, the study addresses maternal sepsis from multiple perspectives: the structural and functional capacity of health systems (WHO QoC), the care experience and whether care aligns with women’s expectations of respectful, inclusive, and equitable treatment (RMC); and delays in accessing care throughout the maternal journey, including patient-related barriers and post-sepsis recovery (4D). This multi-framework approach was necessary due to the complex nature of maternal sepsis, encompassing clinical, structural, and psychosocial dimensions that require a holistic assessment. This integrative approach ensured a nuanced understanding of barriers and opportunities for improving maternal health outcomes. Numerous prioritisation exercises have been conducted on maternal health issues on a global and national scale. However, no studies have focused exclusively on maternal sepsis. For prioritisation exercises, we have demonstrated valuable insights that can be generated by utilising a patient journey for maternal sepsis, particularly in sub-Saharan Africa. Patient journey mapping is increasingly used in person-centred health service design, particularly in association with quality improvement processes, but this technique is rarely used in health research [38–40]. These journeys offer insights into patients’ experiences and provide honest perspectives on the entire care journey, starting from the onset of illness through treatment, hospitalisation, and recovery. This information could supplement the standard hospital audit data used to inform quality improvement initiatives. The journeys have also helped uncover the needs of patients and women for empowerment beyond the urgency of maternal sepsis diagnosis and treatment. The diverse stakeholders (different cadres of healthcare providers, managers, and community representatives) successfully worked together to perform a comprehensive health system assessment by considering what these women went through at every stage of care. The stakeholders found patient journeys valuable for their sensitisation of maternal sepsis, and they recommended that they be used for sepsis awareness among healthcare providers, communities of women and the public.

### Study limitations

First, by holding this workshop in Blantyre, we limited the representation of stakeholders from other regions of the country. We tried to mitigate this by inviting diverse healthcare providers from all cadres and wards through purposive sampling. However, our findings of the interconnected barriers identified across multiple delay stages, RMC, and QoC frameworks are highly likely to be generalisable to other settings. Perspectives on the pragmatic implementation of the proposed interventions will require engagement with central government health officials and policymakers, who were not included as stakeholders at this workshop.

## Conclusion

Patient journeys are a novel tool in Malawi that captures the experience of care and helps identify barriers to care. Engaging multiple stakeholders in the patient journey could guide strategic improvements and targeted interventions to adopt a multifaceted approach to improving the quality of maternal health care in Malawi. It is essential to focus on bolstering the capacity of the maternal healthcare workforce, increasing and upgrading health infrastructure capacity, optimising the quality and performance of the maternal healthcare system, and investing in better medical equipment. Strategic investments in these areas could positively impact the quality of maternity care to reduce preventable sepsis-related maternal morbidity and mortality in Malawi.

## Data Availability

The data supporting this study’s findings are available from the corresponding author, YC, upon reasonable request.
